# On the species status of the root-knot nematode *Meloidogyne mayaguensis* Rammah & Hirschmann, 1988

**DOI:** 10.3897/zookeys.181.2787

**Published:** 2012-04-06

**Authors:** Gerrit Karssen, Jinling Liao, Zhuo Kan, Evelyn YJ van Heese, Loes JMF den Nijs

**Affiliations:** 1 Plant Protection Service, Wageningen Nematode Collection, P.O. Box 9102, 6700 HC Wageningen, The Netherlands; 2 Plant Nematode Lab, College of Environment and Natural Resource, South China Agricultural University, Guangzhou, 510642, PR China

**Keywords:** Junior synonym, *Meloidogyne*, *Meloidogyne enterolobii*, *Meloidogyne mayaguensis*, Nematoda, root-knot nematode, sy- nonymisation

## Abstract

Holo- and paratypes of the root-knot nematodes *Meloidogyne mayaguensis* Rammah & Hirschmann, 1988 and *Meloidogyne enterolobii* Yang & Eisenback, 1983 were morphometrically and morphologically compared. All observed female, male and second-stage juvenile morphometrical and morphological characters are identical for the two studied species. Additionally, contradictions between the original species descriptions were unravelled.

The present study of holo- and paratypes confirms the taxonomical status of *Meloidogyne mayaguensis* as a junior synonym for *Meloidogyne enterolobii*.

## Introduction

In 1983 Yang and Eisenback described the root-knot nematode *Meloidogyne enterolobii* from roots of pacara earpod trees (*Enterolobium contortisiliquum* (Vell.) Morong), on Hainan Island in China. The authors reported severe damage on these pacara earpod trees. In 1988 Rammah and Hirschmann described the root-knot nematode *Meloidogyne mayaguensis* from eggplant (*Solanum melongena* L.) roots, from Puerto Rico. *Meloidogyne mayaguensis* was described by the authors as: “superficially resembles *Meloidogyne enterolobii*”, and reported at the same time “several distinct morphologically features and a unique malate dehydrogenase pattern (N3c)”.

It was Fargette and Braaksma (1990) and Fargette et al. (1996) who reported for the first time on the resistance-breaking behaviour of *Meloidogyne mayaguensis* in Africa and concluded that it is present in both continents of Africa and America. The authors reported (1996) on *Meloidogyne enterolobii*: “*Meloidogyne enterolobii* from China has been described as having the same esterase phenotype as *Meloidogyne mayaguensis*. However it is not known whether their DNA are closely related”. In 2000 Carneiro et al. published esterase and malate dehydrogenase patterns for a Brazilian population of *Meloidogyne mayaguensis*, and detected a different (N1a) malate dehydrogenase pattern. Additionally Blok et al. (2002) published mtDNA results from different *Meloidogyne mayaguensis* populations, including type material from Puerto Rico.

In their comprehensive studies on the characterisation of *Meloidogyne* species from China, with isozymes and mtDNA, Meng et al. (2004) and Xu et al. (2004) included two *Meloidogyne enterolobii* populations from Hainan Island, isolated from the fruit tree Guava (*Psidium guajava* L.). They proved for the first time that *Meloidogyne enterolobii* esterase (VS1-S1) and malate dehydrogenase (N1a) patterns and mtDNA results are identical to reported *Meloidogyne mayaguensis* data, and concluded carefully: “the mtDNA sequence evidence presented here, suggests that *Meloidogyne mayaguensis* could be conspecific with *Meloidogyne enterolobii*”.

In 2005–2006 we compared the available holo- and paratypes of *Meloidogyne enterolobii* and *Meloidogyne mayaguensis*. Meanwhile our Chinese co-authors collected live *Meloidogyne enterolobii* material on Hainan Island at the type locality from the type host and we kindly received live *Meloidogyne mayaguensis* type material from Dr. V. Blok (originating from Dr. M. Fargette). The preliminary isozyme and morphological results were presented by the first author during a Pest Risk Analysis meeting on *Meloidogyne enterolobii* at EPPO in Paris (Anonymous 2008). Additionally this type material of both species was compared at DNA level to *Meloidogyne* sp. from Switzerland and we identified the Swiss population as *Meloidogyne enterolobii* (Kiewnick et al. 2008).

Finally, as again at DNA level no differences were found, the two species were synonymised: “The species *Meloidogyne enterolobii* (syn. *Meloidogyne mayaguensis*)” and “…of *Meloidogyne mayaguensis* (junior synonym of *Meloidogyne enterolobii*)” (Kiewnick et al. 2009).

Although taxonomical not strictly necessary, we present herein a morphological and morphometrical comparison between the holo- and paratype slides of *Meloidogyne mayaguensis* and *Meloidogyne enterolobii*. Additionally we discuss anomalies between the descriptions of *Meloidogyne mayaguensis* and *Meloidogyne enterolobii*.

## Material and methods

Holo- and paratype slides (Table 1) originating from USDA Nematode Collection (USDANC), Beltsville, USA were kindly provided by Dr. Z. Handoo. The type slides are in good condition and includes female holotypes, male allotypes, perineal patterns and second-stage juvenile paratypes. These slides were observed by compound light microscopy (Olympus BH-2 and Zeiss Axio Imager), including Differential Interference Contrast and photographed by Leica DMC-50 digital camera. For the overall morphological and morphometrical comparison between the types we focussed on the most differential and supplementary *Meloidogyne* characters, as described by Jepson (1987) and as previously applied by Karssen (2002). Live type material of both species was propagated and maintained on tomato at the greenhouse of the PPS the Netherlands. This material was studied morphologically (females, males and second-stage juveniles) and used for isozyme electrophoresis (Mdh; EC 1.1.1.37 and Est; EC 3.1.1.1). For details on the preparation of slides and applied electrophoresis method we respectively refer to Karssen (1996) and Karssen et al. (1995).

**Table 1.** *Meloidogyne mayaguensis* and *Meloidogyne enterolobii* holo-, allo- and paratype slides studied, including USDANC codes.

	*Meloidogyne mayaguensis*		*Meloidogyne enterolobii*	
Holotype	1 female	T-428t	1 female	T-360t
Allotype*	1 male	T-429t	1 male	T-361t
Paratype	10 perineal patterns	T-3849p	8 perineal patterns	T-3147p
Paratype	6 males	T-3843p	10 males	T-3149p
Paratypes	25 J2’s	T-3846/7p	25 J2’s	T-3152p

*According to the ICZN rules (4^th^ edition) the allotype concept is no longer valid, and treated herein as a paratype.

## Results and discussion

**Figure 1.**
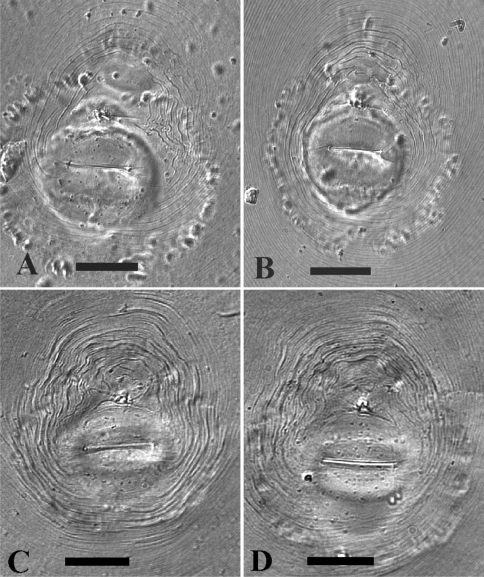
LM photographs of perineal patterns of *Meloidogyne mayaguensis* (**A, B**) and *Meloidogyne enterolobii* (**C, D**). Bar = 25 µm.

**Figure 2.**
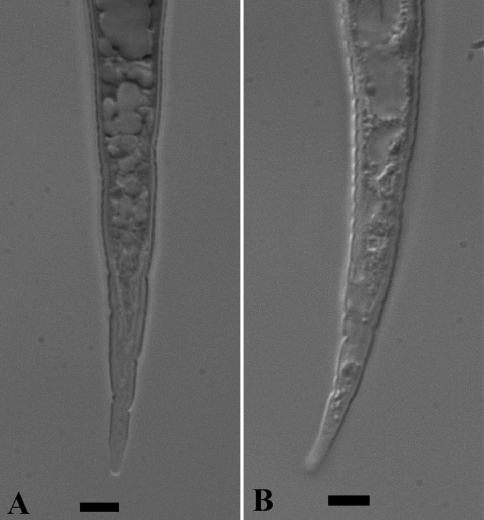
LM photographs of second-stage juvenile tails of *Meloidogyne mayaguensis* (**A**) and *Meloidogyne enterolobii* (**B**). Bar = 5 µm.

See Figure 1 and 2 for LM photographs of female and second-stage juvenile morphological characteristics.

See Table 2–5 for respectively female, male and second-stage juvenile morphological and morphometrical observations.

**Table 2.** Morphological observations of primary female, male and second-stage juvenile characters of *Meloidogyne mayaguensis* and *Meloidogyne enterolobii* holo- and paratypes compared to described data.

**Species**	***Meloidogyne mayaguensis***	***Meloidogyne enterolobii***	
**Character**	**described**		**observed**
**Female**			
Stylet knobs	knobs reniform or transversely elongated, distinctly indented, merging gradually with shaft	knobs set off from shaft, and divided longitudinally by groove so that each knob appears as two	oval, anteriorly often indented, slightly sloping backward to set off
Perineal pattern	round to dorso-ventrally ovoid, dorsal arch rounded, striae fine, single lateral line may occur	oval shaped, with coarse and smooth striae, dorsal arch moderately high to high, often rounded, nearly square in some, lateral lines not distinct	oval shaped, striae mostly fine, dorsal arch rounded to square, weak lateral line(s) sometimes present
**Male**			
Head shape	head not set off, shallowly rounded to truncate, head region high without annulations	head cap high and rounded, head region only slightly set off from body	head cap high and rounded, head region slightly set off, not annulated
Stylet knobs	knobs large, set off from shaft, rounded, sloping backward, dorsal knob base concave	knobs large, rounded, distinctly set off, in some specimens each knob divided longitudinally	knobs large, ovoid to rounded, slightly sloping backwards
**Second-stage juvenile**			
Stylet knobs	knobs small, rounded, set off from shaft, distinctly sloping backward	knobs large, rounded, set off from shaft	knobs ovoid to rounded, slightly sloping backwards
Tail shape	slender, gradually tapering to bluntly rounded tip	very thin, tip broad, bluntly rounded	slender, posterior part nearly straight and parallel, tapering to rounded tip
Hyaline tail part	distinctly set off, often containing small fat droplet at tip	clearly defined, a few fat droplets may occur in terminus	anterior part not clearly delimitated

### Females

The important morphological characters, like female stylet knob and perineal pattern shape do not differ between the species, as can already be observed by comparing the original illustrations between *Meloidogyne mayaguensis* and *Meloidogyne enterolobii* (see original descriptions respectively Fig. 2 A–D & Fig. 3 A–D). This perineal pattern type is not species specific within the genus *Meloidogyne* and can best be marked as typical for many species within the *Meloidogyne incognita*-group, including the observed variation within the dorsal part. Additionally we observed a relatively large tail remnant area, free of any striae, just above the covered anus (Fig. 1 A–D). Also the observed stylet knob position variation, slightly sloping backward to set off from the shaft, is a common *Meloidogyne* feature. Strangely this variation is also clearly visible in the SEM photographs of excised female stylets of *Meloidogyne mayaguensis* (see original description, Fig. 3 A-C), but not described. With the light microscope one can observe a weak longitudinal indention, for both species, in the female stylet knobs at the anterior side. The reported differences “not divided so conspicuously as those of *Meloidogyne enterolobii*” as mentioned in the *Meloidogyne mayaguensis* description (see diagnosis original description), was not confirmed by our observations. Also the described position of one of the *Meloidogyne mayaguensis* stylet knobs “the dorsal knob is slightly sloping posteriad in lateral view” was not observed by us.

### Males

The male head shape for *Meloidogyne mayaguensis* is described as “not set off”, while a slightly set off head region was observed as described for *Meloidogyne enterolobii*. Comparing the original SEM pictures of the head for *Meloidogyne mayaguensis* and *Meloidogyne enterolobii* (see original descriptions respectively Fig. 6 A–D & 5 A,B) shows clearly not any differences in head morphology. Also the male stylet knobs have been SEM studied for the original descriptions (Fig. 3 E, F & Fig. 6 B) of both species. Large oval to rounded shaped knobs, slightly sloping backwards are clearly visible. This was also observed by LM for both species, however described as “rounded and set off” for *Meloidogyne enterolobii* and “set off from the shaft, rounded, sloping backward” for *Meloidogyne mayaguensis*. The later description of the knobs is rather odd, i.e. set off and sloping backward at the same time!The same results were described and observed for the second-stage juvenile knobs for both species.

### Second-stage juveniles

The second-stage juvenile stylet knob size is described as small for *Meloidogyne mayaguensis* and large for *Meloidogyne enterolobii*. We indeed observed a larger size variation for *Meloidogyne enterolobii* stylet knob width (2.5 – 4.0 µm) compared to *Meloidogyne mayaguensis* (2.2 – 2.9 µm). However when observing live second-stage juveniles, the same large stylet knob width variation was observed for both species.

As for the males, the published SEM second-stage juvenile head shape is absolute identical for *Meloidogyne mayaguensis* and *Meloidogyne enterolobii* (see original descriptions respectively Fig. 7 A–D & Fig. 8 A, B). The tail is distinctly tapering and in the posterior tail (roughly the hyaline tail part) nearly straight and running parallel for both second-stage juvenile paratypes. Also, for both species the hyaline tail part is described as “distinctly set off” or “clearly defined”. We observed for both species however not a clearly anterior delimitated hyaline tail part, in fact the body content runs deep into the hyaline tail part (Fig 2 A, B), as comparable to *Meloidogyne hapla* (Karssen, 2002). The second-stage juvenile drawings for both species descriptions (Fig. 4 E, F & Fig. 7 E–F) show a clearly delimitated anterior hyaline tail part, while the original photographs (Fig. 5 F, G & Fig. 9 B) do not show this at all. The fact that both descriptions did not include the hyaline tail measurements (a standard procedure), suggest strongly that the hyaline tail part is not clearly defined. Also in live second-stage juveniles we did not observe a clearly defined hyaline tail part (Table 2).

### Morphometrics

The morphometrical characters between the types of *Meloidogyne mayaguensis* and *Meloidogyne enterolobii* (Table 3–5), are comparable for the described and observed data, i.e. all mean data are the same or at least within the calculated range. Body length and body width data are generally slightly smaller when comparing observed to described data, this is a well known effect due to a slight shrinking of the nematode body within permanent slides. For *Meloidogyne enterolobii* males we noticed however an unusual difference in greatest body width between the described 42.3 µm (37–48 µm) and observed 32.0 µm (24–39) µm data. The differences can not only be explained due to a shrinking effect, particularly as the observed greatest body width data agrees with the observed data for *Meloidogyne mayaguensis*. Also for the *Meloidogyne enterolobii* female holotype unexplainable differences were noticed between described and observed data for the DGO (3.7 µm versus 4.8 µm) and stylet length (13.4 µm versus 14.7 µm).

**Table 3.** Morphometrical (in µm) observations (mean, SD & range) of female *Meloidogyne mayaguensis* and *Meloidogyne enterolobii* holo- (single female) and paratypes (perineal patterns) compared to described data.

**Species**	***Meloidogyne mayaguensis***		***Meloidogyne enterolobii***	
**Character**	**description**	**observed**	**description**	**observed**
**Holotype** (N)	1	1	1	1
Body length	720	674	667	693
Body width	570	576	415	462
Neck length	190	168	265	262
Neck width	160	169	--	--
DGO	6.2	6.4	3.7	4.8
Excretory pore tohead end	46.4	45.8	44.8	64.0
Stylet length	15.1	15.7	13.4	14.7
Stylet knob height	2.2	2.0	2.7	2.3
Stylet knob width	4.4	4.5	4.3	4.5
**Paratypes** (N)	35	10	20	8
Interphasmidial dist.	23.2 ± 2.5 (18.1–29.6)	28.8 ± 3.7 (24.3–33.3)	30.7 ± 4.8 (22.2–42.0)	33.5 ± 7.6 (22.4–41.9)
Vulval slit length	26.1 ± 1.9 (20.9–30.4)	27.0 ± 1.4 (25.0–29.4)	28.7 ± 2.0 (25.3–32.4)	28.0 ± 1.0 (25.9–29.1)
Vulva-anus distance	18.4 ± 1.5 (12.7–21.1)	21.4 ± 3.1 (17.0–27.1)	22.2 ± 1.8 (19.7–26.6)	23.4 ± 1.6 (21.1–26.2)
DGO	4.8 ± 0.8 (3.5–6.7)	–	4.9 ± 0.8 (3.7–6.2)	–
Excretory pore to head end	48.2 ± 13.6 (25.9–86.6)	–	62.9 ± 10.5 (42.3–80.6)	–
Stylet length	15.8 ± 0.8 (13.8–16.8)	–	15.1 ± 1.4 (13.2–18.0)	–

**Table 4.** Morphometrical (in µm) observations (mean, SD & range) of male *Meloidogyne mayaguensis* and *Meloidogyne enterolobii* paratypes compared to described data.

**Species**	***Meloidogyne mayaguensis***		***Meloidogyne enterolobii***	
**Character**	**description**	**observed**	**description**	**observed**
N	30	7	20	11
Body length	1503 ± 142 (1175–1742)	1431 ± 63 (1337–1496)	1600 ± 160 (1349–1913)	1230 ± 316 (865–1667)
Greatest body width	37.8 ± 3.1 (32.2–44.4)	34.5 ± 1.9 (32.0–37.4)	42.3 ± 3.6 (37.0–48.3)	32.0 ± 6.0 (23.7–39.2)
Stylet length	22.9 ± 0.8 (20.7–24.6)	22.1 ± 0.7 (20.8–23.0)	23.4 ± 1.0 (21.2–25.5)	21.5 ± 1.7 (19.2–23.4)
Stylet knob height	3.0 ± 0.3 (2.4–3.7)	3.2 ± 0.3 (2.6–3.4)	3.3 ± 0.3 (2.6–3.9)	2.5 ± 0.3 (2.1–3.2)
Stylet knob width	5.0 ± 0.3 (4.3–5.6)	5.3 ± 0.5 (4.5–5.8)	5.4 ± 0.3 (4.5–5.8)	4.5 ± 0.6 (3.5–5.0)
DGO	4.1 ± 0.4 (3.3–5.0)	4.1 ± 0.7 (3.2–5.1)	4.7 ± 0.4 (3.7–5.3)	4.7 ± 0.6 (3.7–5.8)
Excretory pore to head end	166.4 ± 8.8 (147.2–180.8)	158.6 ± 14.9 (132.5–177.9)	178.2 ± 11.2 (159.7–206.2)	155.8 ± 22.3 (129.9–199.7)
Spicule length	28.3 ± 1.5 (24.4–31.3)	29.0 ± 2.4 (25.6–32.3)	30.4 ± 1.2 (27.3–32.1)	28.0 ± 1.1 (26.2–29.4)
Gubernaculum length	7.1 ± 0.6 (6.1–9.3)	7.5 ± 1.0 (6.4–9.0)	6.2 ± 1.0 (4.8–8.0)	6.5 ± 0.8 (6.1–8.0)
Tail length	14.3 ± 1.1 (11.3–16.3)	13.0 ± 1.1 (10.9–14.7)	12.5 ± 2.2 (8.6–20.2)	11.9 ± 1.2 (10.2–13.4)
A	39.9 ± 3.9 (31.1–49.6)	41.6 ± 2,9 (37.2–44.7)	37.9 ± 3.2 (34.1–45.5)	38.1 ± 4.0 (30.0–43.4)
C	105.7 ± 10.0 (85.8–124.3)	110.5 ± 10.8 (98.5–133.7)	131.6 ± 24.2 (72.0–173.4)	103.2 ± 23.7 (71.4–135.9)

**Table 5.** Morphometrical (in µm) observations (mean, SD & range) of second-stage juvenile *Meloidogyne mayaguensis* and *Meloidogyne enterolobii* paratypes compared to described data.

**Species**	***Meloidogyne mayaguensis***		***Meloidogyne enterolobii***	
**Character**	**description**	**observed**	**description**	**observed**
N	35	25	30	25
Body length	454 ± 28 (390–528)	420 ± 21 (386–456)	437 ± 17 (405–473)	408 ± 18 (380–442)
Greatest body width	14.7 ± 0.5 (13.8–15.8)	13.9 ± 0.7 (13.1–15.4)	15.3 ± 0.9 (13.9–17.8)	14.8 ± 2.1 (11.0–18.0)
Body width at anus	10.9 ± 0.5 (10.2–12.2)	9.8 ± 0.6 (9.0–11.2)	–	9.8 ± 0.9 (8.0–11.0)
Stylet length	11.6 ± 0.3 (11.1–12.2)	11.5 ± 0.4 (10.9–12.1)	11.7 ± 0.5 (10.8–13.0)	11.3 ± 0.7 (10.5–13.0)
Stylet base to head end	15.2 ± 0.3 (14.8–15.8)	15.4 ± 0.3 (14.7–16.0)	–	15.0 ± 0.7 (14.0–16.0)
Stylet knob height	–	1.5 ± 0.1 (1.2–1.7)	1.6 ± 0.1 (1.3–1.8)	1.8 ± 0.3 (1.5–2.0)
Stylet knob width	–	2.5 ± 0.2 (2.2–2.9)	2.9 ± 0.3 (2.4–3.4)	3.0 ± 0.4 (2.5–4.0)
DGO	3.9 ± 0.2 (3.3–4.3)	3.7 ± 0.4 (3.2–4.2)	3.4 ± 0.3 (2.8–4.3)	3.8 ± 0.3 (3.0–4.5)
Excretory pore to head end	87.6 ± 3.3 (79.9–97.9)	88.3 ± 3.0 (83.5–95.3)	91.7 ± 3.3 (84.0–98.6)	80.8 ± 4.4 (70.0–88.0)
Tail length	54.4 ± 3.6 (49.2–62.9)	54.2 ± 2.7 (48.7–58.5)	56.4 ± 4.5 (41.5–63.4)	52.1 ± 3.4 (45.0–57.0)
a	30.9 ± 1.9 (26.4–34.7)	30.1 ± 1.6 (26.9–32.8)	28.6 ± 1.9 (24.0–32.5)	28.0 ± 3.7 (23.3–34.6)
c	8.3 ± 0.4 (7.0–9.2)	7.8 ± 0.3 (7.1–8.4)	7.8 ± 0.7 (6.8–10.1)	7.9 ± 0.6 (7.0–9.0)
Excretory pore (%)	19.4 ± 1.0 (17.8–22.3)	21.1 ± 0.9 (19.2–22.7)	–	19.8 ± 1.1 (17.6–21.9)

The described and discussed *Meloidogyne mayaguensis* differences (see diagnosis original description)within the female perineal pattern for the interphasmidial distance, vulval slit length and vulva-anus distance is not confirmed by our observations. All these measurements are within the observed range. Perineal pattern measurements are generally highly variable and a logical reason for Jepson (1987) not to list this type of data when discussing differential characters for the genus *Meloidogyne*.

### Reproduction and cytogenetics

The two species descriptions report also on the mode of reproduction and number of chromosomes, both reproduce by mitotic parthenogenesis (= apomixes) and have a somatic chromosome number of 2n = 44–45 for *Meloidogyne mayaguensis* and 2n = 44–46 for *Meloidogyne enterolobii*. In conclusion, both species have the same mode of reproduction and somatic chromosome number.

### Host plants

Additionally, both species descriptions report in their introduction part some hosts, i.e. they both previously applied the North Carolina differential host test (Hartman and Sasser 1985). Both species showed the same positive host response for tobacco, pepper, watermelon and tomato and no host response on peanut. Beside this, *Meloidogyne mayaguensis* did not infest cotton, while *Meloidogyne enterolobii* moderately infested cotton. As the details of the previously applied host tests have not been described in the material and method part of the species descriptions, we can not explain the reported host response differences on cotton for *Meloidogyne mayaguensis* and *Meloidogyne enterolobii*. Interesting is the *Meloidogyne mayaguensis* study by Brito et al. (2004) with four isolates from Florida (USA). All four isolates, maintained on tomato, reproduced also on cotton, tobacco, pepper and watermelon but not on peanut, i.e. identical to the published results for *Meloidogyne enterolobii*.

### Isozymes

The observed esterase (VS1-S1 type) and malate dehydrogenase (N1a type) isozyme patters are identical for both species and agrees with previous results (Carneiro et al. 2000; Xu et al. 2004).

## Conclusion

In conclusion, the holo- and paratype material of *Meloidogyne mayaguensis* and *Meloidogyne enterolobii* is morphological and morphometrical identical and it confirms the taxonomical status of *Meloidogyne mayaguensis* as a junior synonym for *Meloidogyne enterolobii*.
